# Altered cleavage plane orientation with increased genomic aneuploidy produced by receptor-mediated lysophosphatidic acid (LPA) signaling in mouse cerebral cortical neural progenitor cells

**DOI:** 10.1186/s13041-020-00709-y

**Published:** 2020-12-14

**Authors:** Whitney S. McDonald, Kyoko Miyamoto, Richard Rivera, Grace Kennedy, Beatriz S. V. Almeida, Marcy A. Kingsbury, Jerold Chun

**Affiliations:** 1 Sanford Burnham Prebys Medical Discovery Institute, 10901 N Torrey Pines Rd, La Jolla, CA 92037 USA; 2 The Scripps Research Institute, La Jolla, CA 92037 USA

**Keywords:** LPA, Lysophospholipid, GPCR, Neural progenitor cells, NPC, Adherens junctions, Somatic genomic mosaicism, Aneuploidy, Cleavage plane, Hydrocephalus

## Abstract

The brain is composed of cells having distinct genomic DNA sequences that arise post-zygotically, known as somatic genomic mosaicism (SGM). One form of SGM is aneuploidy—the gain and/or loss of chromosomes—which is associated with mitotic spindle defects. The mitotic spindle orientation determines cleavage plane positioning and, therefore, neural progenitor cell (NPC) fate during cerebral cortical development. Here we report receptor-mediated signaling by lysophosphatidic acid (LPA) as a novel extracellular signal that influences cleavage plane orientation and produces alterations in SGM by inducing aneuploidy during murine cortical neurogenesis. LPA is a bioactive lipid whose actions are mediated by six G protein-coupled receptors, LPA_1_–LPA_6_. RNAscope and qPCR assessment of all six LPA receptor genes, and exogenous LPA exposure in LPA receptor (*Lpar*)-null mice, revealed involvement of *Lpar1* and *Lpar2* in the orientation of the mitotic spindle. *Lpar1* signaling increased non-vertical cleavage in vivo by disrupting cell–cell adhesion, leading to breakdown of the ependymal cell layer. In addition, genomic alterations were significantly increased after LPA exposure, through production of chromosomal aneuploidy in NPCs. These results identify LPA as a receptor-mediated signal that alters both NPC fate and genomes during cortical neurogenesis, thus representing an extracellular signaling mechanism that can produce stable genomic changes in NPCs and their progeny. Normal LPA signaling in early life could therefore influence both the developing and adult brain, whereas its pathological disruption could contribute to a range of neurological and psychiatric diseases, via long-lasting somatic genomic alterations.

## Introduction

The vertebrate brain is composed of cells having distinct genomes that produce a complex genomic mosaic, which appears to arise at multiple points of brain development and maturity, including amongst neural progenitor cells (NPCs) during neurogenesis [1, 2]. The cellular fate of NPCs in mammalian brain development is in part governed by the mitotic spindle and cleavage plane orientation at cell division. Disruption of the cleavage plane orientation can lead to abnormal cortical cytoarchitecture and other developmental phenotypes [3–8]. The predominant cleavage plane orientation of radial glial progenitor cells (RGPs) is vertical (*i.e.* perpendicular) to the ventricular surface, which expands the progenitor pool through symmetric division [9, 10]. “Non-vertical” oblique or horizontal cleavage plane orientation occurs just prior to neurogenesis when RGPs differentiate into intermediate progenitors or NPCs [6, 9, 11, 12]. This asymmetric, non-vertical cleavage is highly correlated with neurogenic division [6, 9, 11, 12] and leads to an increase in neuronal differentiation [3–8].

Multiple intracellular or membrane components, such as centrosomes, microtubule-associated proteins, basolateral proteins, G proteins, and adherens junctions (AJs), are regulators of cleavage plane orientation and mitotic spindle position. Within these components, further heterogeneity exists, as underscored by AJs that are composed of cell-to-cell adhesion proteins, such as beta-catenin and N-cadherins, that interact with the Par3 complex (Par3, Par6, and aPKC) to maintain apicobasal polarity of the neuroepithelium, orient the mitotic spindle, and regulate the cleavage plane [3–7, 13, 14]. Mutations that disrupt the mitotic spindle assembly or polarity proteins can also lead to atypical NPC mitosis, which is associated with depletion of progenitor pools and altered neurogenesis [15, 16].

Concomitant with effects on the mitotic spindle is chromosomal segregation that classically produces two daughter cells after cell division, each of which contains an identical 2N number of chromosomes. However, NPC production, even during normal development, can also produce aneuploid daughter cells [1, 15–17]. Aneuploidy is defined as the gain and or loss of chromosomes from the euploid complement, which in mice is 40 chromosomes. Aneuploidy is also a form of DNA copy number variation (CNV) that contributes to SGM and the range of genomic alterations observed within individual brain cells. Beyond aneuploidy and CNVs, SGM includes Line1 elements, insertions, deletions and single nucleotide variations (SNVs) [18]. Aneuploid neural cells show altered transcriptomes [19], cell death, and cell survival [20–22], can be functionally integrated into the brain’s circuitry [23, 24], and can contribute to neurological disease [19, 25–27]. These genomic changes arise somatically; however, it remains unclear whether they are purely cell-autonomous stochastic changes or include non-cell-autonomous extracellular signaling.

A signaling molecule that can influence NPCs is lysophosphatidic acid (LPA), a small phospholipid with diverse functions mediated by six known G protein-coupled receptors (GPCRs): *Lpar1-6* [28, 29]. LPA is present in various biological fluids and tissues including the cerebrospinal fluid [30] and the brain parenchyma [31, 32]. The importance of *Lpar1* in brain development has been demonstrated in reports of *Lpar1*-null mutant mice that exhibit defective neurogenesis in the developing cortex and adult hippocampus, along with anxiety-like behavior and spatial memory deficits [33–35, 61]. Gain-of-function studies have shown *Lpar1* and *Lpar2*-mediated cortical growth and neuronal differentiation [36, 37]. In the developing mouse fetus, ventricular exposure to LPA disrupts the cortical cytoarchitecture and recapitulates neurodevelopmental disease phenotypes such as hydrocephalus [38, 39] and neuropsychiatric abnormalities [40, 41]. These long-term effects suggest that LPA exposure during development can not only produce acute cellular changes, but might alter cleavage plane orientation of dividing cells to generate aneuploidy and, possibly, other genome-altering mechanisms. Here we examine effects of LPA signaling on NPC cleavage plane orientation, cell fate, and SGM manifesting as aneuploidy.

## Materials and methods

### Animal use

Animal use protocols were approved by the Institutional Animal Care and Use Committee at The Scripps Research Institute and Sanford Burnham Prebys Medical Discovery Institute and conformed to National Institutes of Health guidelines and public law. Embryonic day (E) 13.5 timed pregnant C57BL/J6 mice or *Lpar1*/*Lpar2* mutant mice on a mixed background of C57BL/6J and 129/SvJ [42] were used for all in vivo and ex vivo studies.

### In vivo LPA ventricle injection

E13.5 embryos were injected in utero with LPA 18:1 (Oleoyl-LPA, Avanti Polar Lipids) in 0.01% fatty-acid-free bovine serum albumin (FAFBSA; Roche) at an effective concentration of 1.4 μM [43]. Timed pregnant (E13.5) mice were anesthetized with Nembutal (50 mg/kg) or isoflurane (1–3%) and placed prone on a sterile drape and a warmed delta-phase heating pad. The incision site was shaved and cleansed with alternating swabs of povidone-iodine solution and 70% ethanol. The uterine horns were exposed using a midline ¾ inch sagittal incision through the body wall, giving access to the peritoneal cavity. The cerebral ventricles in the fetus were visualized through the uterine wall by direct illumination with a fiber optic light source and binocular dissecting scope. Three µl of LPA or vehicle (0.01% FAFBSA) solution were injected into the ventricles using a micro-syringe (36 gauge). After injection, the uterus was returned to the peritoneal cavity, the body wall was closed with non-wicking sutures and the surgical site was swabbed with povidone-iodine solution to prevent infection. Aseptic technique was followed throughout the surgical procedure. Embryonic brains were isolated 6 h after LPA injection.

### Cortical hemisphere cultures

To create ex vivo cortical hemisphere cultures, E13.5 timed-pregnant C57BL/6J mice or *Lpar1*/*Lpar2* mutant mice on a mixed background of C57BL/6J and 129/SvJW were euthanized by isoflurane overdose followed by cervical dislocation, and embryos were removed. Embryos from *Lpar1*/*Lpar2* mutants were genotyped by PCR [42] using genomic DNA isolated from the tail. Brains of embryos were dissected out in 0.1 M PBS (pH 7.4). The cortical hemispheres of each brain were separated along the midline and placed in 50 ml conical tubes containing 2 ml of serum-free ex vivo culture medium (Opti-MEM I, Gibco) supplemented with 20 mM d-glucose (Sigma), 55 µM β-mercaptoethanol (Gibco) and 1% penicillin/streptomycin/l-glutamine (Gibco) saturated with 5% CO_2_. One hemisphere was cultured in medium containing 1 µM LPA in 0.1% FAFBSA (Roche), and the other hemisphere was cultured in control medium containing 0.1% FAFBSA or 2 mM EGTA in 0.01% FAFBSA. Hemispheres were cultured at 37 ºC for 1–17 h, with agitation at 65 r.p.m. At the end of the culture period, hemispheres were processed for metaphase spread analysis or fixed in 4% paraformaldehyde (PFA) in PBS, cryoprotected in 30% sucrose in PBS, embedded in Tissue-Tek (Sakura), and rapidly frozen on powdered dry ice for immunohistochemistry.

### Metaphase spreads

The nuclear chromosome content of cortical progenitors was assessed by isolating cortices from E13.5 embryos 6 h after intraventricular injection of LPA or vehicle (0.01% BSA) or 12 h after LPA exposure for ex vivo cultures. Cortices were then dissociated and prepared in single-cell suspensions. Progenitor cells were arrested in metaphase in a serum-free colcemid solution (100 ng/ml) at 37 °C for 3 h. Cell membranes were then swollen with a 75 mM KCl solution and fixed in 3:1 methanol/glacial acetic acid. 90–100 metaphase cells were analyzed per treatment group using a Zeiss Axio Imager.M2fluorescence microscope.

### Immunohistochemistry and immunocytochemistry

The following antibodies were used in this study: the mouse monoclonal antibodies, anti-Nestin (clone Rat 401; BD Biosciences) and anti-β-catenin (clone 14/Beta-Catenin; BD Biosciences); the rabbit polyclonal antibodies, anti-Tuj1 (Covance), anti-N-cadherin (Calbiochem), anti-Par3 (Milllipore) and anti-Pax6 (BioLegend); and a chicken polyclonal antibody, anti-Tbr2 (Millipore). Primary antibodies were detected with AF488-conjugated donkey anti-mouse antibody (Molecular Probes) or Cy3-conjugated donkey anti-rabbit antibody (Millipore). DAPI (Fluka) or TO-PRO 3 (Molecular Probes) was used for nuclear counterstaining. PFA-fixed cryosections or dissociated cells were blocked with PBS containing 5% normal serum (Vector) and 0.1% Triton X-100. Primary and secondary antibodies were diluted in PBS containing 1% normal serum and 0.1% Triton X-100. Sections were pretreated with microwave antigen retrieval in 0.01 M sodium citrate (pH 6.0) and 0.05% Tween 20 or Diva Decloaker (Biocare Medical). Images were collected using an Axio Imager D1 fluorescent microscope (Zeiss) with Axioimage 4.7.1 software (Zeiss), and prepared with Adobe Photoshop Elements 3.0 (Adobe Systems). Confocal images were collected using a FluoView 500 laser-scanning confocal (Olympus) mounted on a BX61 microscope (Olympus), and processed using MetaMorph (Molecular Devices), NIH ImageJ 10.2 and Adobe Photoshop Elements 3.0 software.

### RNAscope

RNAscope was performed according to the manufacturer’s instructions on 5–6 µm sections from E13.5 formalin-fixed paraffin-embedded brains. Briefly, sections were treated with H_2_O_2_ for 10 min to block endogenous peroxidase, then stained with RNAscope 2.5 HD Assay—BROWN (Advanced Cell Diagnostics, Inc.) to amplify and detect LPA receptor RNA signals. The sections were then counterstained with Gills Hematoxylin to visualize brain structures. Images were acquired on an Axio Imager D1 fluorescent microscope (Zeiss) with Axioimage 4.7.1 software (Zeiss).

### Analyses of cleavage plane orientation

Apical progenitor cell cleavage plane orientation was determined using DAPI-stained cryosections (14–20 µm) or HE-stained paraffin sections (10 µm). Anaphase cells on the apical surface of the dorsal cortex were examined and classified into three groups (vertical, 60º–90º; oblique, 30º–60º; horizontal, 0º–30º) based on the cleavage plane angle relative to the ventricular surface.

### Pair-cell analysis

Cortices were dissected from E13.5 embryos, with careful removal of meninges and choroid plexus, and triturated into single cells by gentle pipetting with a P1000 tip in serum-free dissociation culture medium: DMEM (Gibco) supplemented with B27 supplement without retinoic acid (Gibco), N2 supplement (Gibco), 10 ng/ml recombinant human FGF-basic (PeproTech), non-essential amino acids (Gibco), sodium pyruvate (Gibco), and 1% penicillin/streptomycin/glutamine. Cells were dispersed at a clonal density on a Lab-Tek chamber glass slide (Nunc) coated with Cell-Tak (Corning) and cultured in 0.1% FAFBSA medium with or without 1 µM LPA. Immediately after plating and the addition of LPA, cells were monitored with a Yokogawa CSU-10 spinning-disk confocal mounted onto an Olympus IX70 microscope. Images were collected with MetaMorph software (v 7.1). Cells were kept at 37 ºC and continuously supplied with 5% CO_2_ and 21% O_2_ during imaging. DIC images were acquired every 2 h using a 10× objective. After 20 h, cells were fixed with 4% PFA in PBS, and immunolabeled for nestin and Tuj1. Time-lapse DIC and fluorescent images in the same field were prepared using MetaMorph and stacked using NIH ImageJ 10.2 software. Cells that underwent mitosis during imaging were chosen and classified into three groups: P–P (2 nestin-positive daughters), P–N (1 nestin-positive and 1 Tuj1-positive daughter) and N–N (2 Tuj1-positive daughters).

### Quantitative real-time PCR

Total RNA was isolated from E13.5 cerebral cortex or from E13.5 cortical cells cultured in serum-free media for 20 h using Trizol reagent (Thermo Fisher Scientific) according to the manufacturer’s protocol. The RNA was then treated with DNAse, primed with oligo (dT), and cDNA was synthesized using SuperScript II reverse transcriptase (Thermo Fisher Scientific). qPCR was carried out using a Bio-Rad CFX384 real-time PCR detection system, TaqMan probes (Applied Biosystems), and Taqman Fast Advanced Master Mix (Applied Biosystems). Transcripts for mouse *Lpar1-6* and β-actin were detected with the following Taqman probes, respectively: Mm01346925_m1, Mm00469562_m1, Mm00469694_m1, Mm01228533_m1, Mm02621109_s1, Mm00613058_s1, and Mm02619580_g1. Samples were measured in triplicate and the mouse β-actin probe was used for normalization to determine the relative expression of each gene by the 2^−ΔCT^ method [44].

### Statistical analysis

The numbers of animals per group are indicated in the figure legends. The data are reported as the mean ± SEM, p value. All statistical analyses were performed on Graphpad Prism (version 8.1.0). Statistical differences were determined using ANOVA with Dunnett's post-hoc test for multiple comparisons for normally distributed data and the Kruskal–Wallis test with Dunn’s post-hoc test for skewed data.

## Results

### LPA receptor signaling modulates cleavage plane orientation of apical progenitors in vivo

**Fig. 1:**
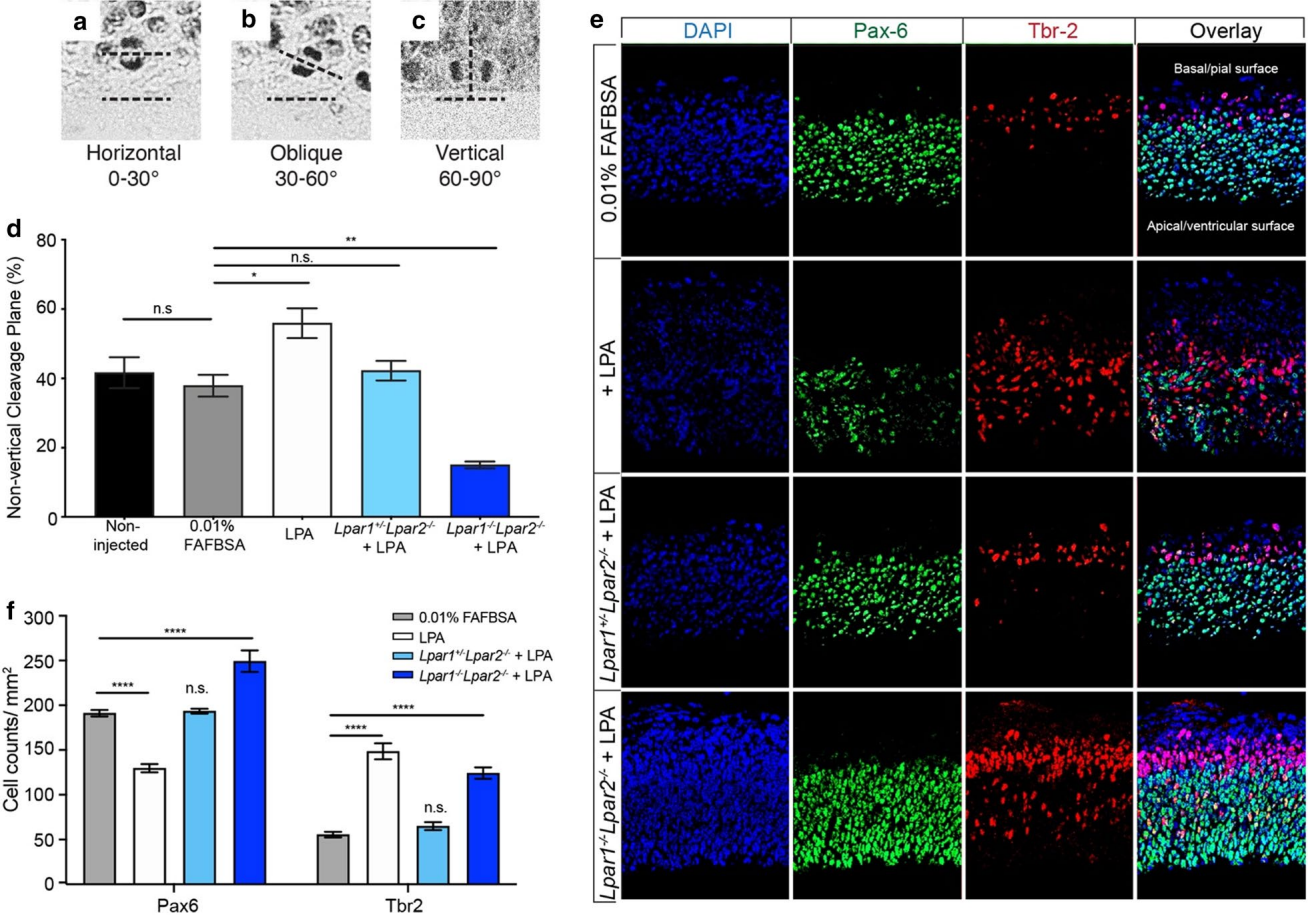
LPA receptor signaling increases the frequency of non-vertical cleavage planes and alters pro-neurogenic cell fates in vivo. Representative images of apical mitotic progenitor cells at anaphase with **a** a vertical cleavage plane (60°–90°), **b** an oblique cleavage plane (30°–60°), and **c** a horizontal cleavage plane (0°–30°). Cells were classified into these three groups based on the angle of the mitotic cleavage plane relative to the ventricular surface. **d** Percentage of cells with non-vertical cleavage planes following in vivo injection of LPA (1.4 µM) or vehicle (0.01% BSA) into the lateral ventricles at E13.5. Lpar1 signaling increased the percentage of cells having a non-vertical cleavage plane. Black bar: non-injected (n = 5); gray bar: vehicle (n = 4); white bar: LPA injection (n = 5); light blue bar: LPA injection into *Lpar1*^+/−^*Lpar2*^−/−^ mutant mice (n = 3); and dark blue bar: LPA injection into *Lpar1*^−/−^*Lpar2*^−/−^ mice (n = 3). Methods: brains were fixed 6 h after injection, embedded in paraffin, sectioned at 10 µm and stained with hematoxylin; the cleavage plane angle of anaphase cells was measured throughout the left ventricle in 30 µm increments. Data shown as mean ± SEM. Statistical significance determined by ANOVA with Dunnett’s post-hoc test for multiple comparisons against vehicle-injected controls; n.s. = not significant (P ≥ 0.05), *P = 0.033, **P = 0.0021, ***P = 0.0001). Scale bar 10 µm. **e** LPA signaling displaces neural progenitor cell population. Representative images comparing LPA effects, relative to vehicle, on progenitor cells (Pax6, green), intermediate progenitor cells (Tbr2, red) and nuclear staining (DAPI, blue) in the cortex of wildtype, *Lpar1*^+/−^*Lpar2*^−/−^, and *Lpar1*^−/−^*Lpar2*^−/−^ E13.5 mice. Overlaid images (right-most panels) illustrate layer distinctions. **f** LPA signaling increases the number of Tbr2+ cells. Methods: Total cortical neural progenitor cells (Tbr2 + cells) were counted in 50 μm sagittal sections of E13.5 cortices, 550–950 μm lateral (9 sections analyzed per treatment group). Vehicle-injected wildtype mice (n = 3) were compared with 1.4 μM LPA-injected wildtype (n = 3), *Lpar1*^+/−^*Lpar2*^−/−^ (n = 3), and *Lpar1*^−/−^*Lpar2*^−/−^ (n = 3) mice. Statistical significance determined by ANOVA with Dunnett’s post-hoc test for multiple comparisons against vehicle-injected control. Data shown as mean ± SEM., n.s. = not significant (P ≥ 0.05), *P = 0.033, **P = 0.0021, ***P = 0.0001; unpaired *t* tests)

We hypothesized that LPA exposure during development may alter the cleavage plane of NPCs prior to neurogenesis. To test this idea in vivo, E13.5 embryos were exposed to a pathologically relevant concentration of LPA (1.4 µM) [31] by intraventricular injection, and the cleavage plane of apical progenitors was assessed 6-h later. We only assessed cells that had completed metaphase since the cleavage plane orientation of an apical mitotic cell is stable once it enters anaphase [45]. Cleavage plane orientation was classified based on the angle of orientation relative to the ventricular surface, such that the cells fell into three groups: vertical (60º–90º), oblique (30º–60º), and horizontal (0º–30º) (Fig. 1a–c). There was a significant increase in the percentage of cells undergoing non-vertical cleavage following LPA injection (55.9% ± 4.3), compared with vehicle-injected controls (37.9% ± 3.14) (Fig. 1d). To determine if these effects are mediated by specific LPA receptor(s), and to reduce possible in vivo compensation by *Lpar2* for a loss of *Lpar1*, we compared *Lpar1*^+/−^ and *Lpar1*^−/−^ littermates in the *Lpar2*-null background. The LPA-induced increase in non-vertical cleavage was absent in *Lpar1*^+/−^*Lpar2*^−/−^ mice (42.2% ± 2.8), whereas *Lpar1*^−/−^*Lpar2*^−/−^ mice (15.0% ± 1.0) had significantly decreased non-vertical cleavage relative to vehicle control (Fig. 1d).

Cleavage plane orientation dictates daughter cell fate. To determine the effect of LPA signaling on cortical cell fates in vivo, brain sections from LPA and vehicle-injected E13.5 mice were stained with: (1) paired box protein-6 (Pax6), a transcription factor expressed by radial glial cells and progenitor cells migrating through the cortical layers; and (2) T-box brain protein 2 (Tbr2), a marker for intermediate neural progenitors that are typically localized to the cortical plate during early- to mid-neurogenesis. We observed stereotypical staining for both Pax6 and Tbr2 in vehicle-injected brains (Fig. 1e). However, LPA injection decreased Pax6 expression (129.7 ± 4.55), whereas Tbr2 expression increased (148.55 ± 8.91) compared with controls (Fig. 1e, f). Further, the Tbr2+ cell population was abnormally positioned in the ventricular and subventricular zones (Fig. 1e), which correlates with the observed increase in non-vertical cleavage after LPA exposure (Fig. 1d). In *Lpar1*^+/−^*Lpar2*^−/−^ mutant mice exposed to LPA, Pax6 + (193.44 ± 2.69) and Tbr2+ cell counts (65.0 ± 4.45) were not significantly different from controls. However, in *Lpar1*^*−/−*^*Lpar2*^−/−^ mutant mice exposed to LPA, Pax6+ cells were observed throughout the ventricular and subventricular zones and were significantly increased (249.22 ± 12.07) compared to vehicle controls. Notably, there was also an observed increase in the number of Tbr2+ cells (124.22 ± 6.36) at the cortical plate and some increased expression in the subventricular and ventricular zones (Fig. 1e, f). Taken together, these data suggest that cortical neuron production is prematurely enhanced in E13.5 mice exposed to LPA, and that cell fate was controlled—at least in part—by LPA signaling through both LPA_1_ and LPA_2_.

### LPA receptors are expressed at the ventricular surface during neurogenesis

**Fig. 2:**
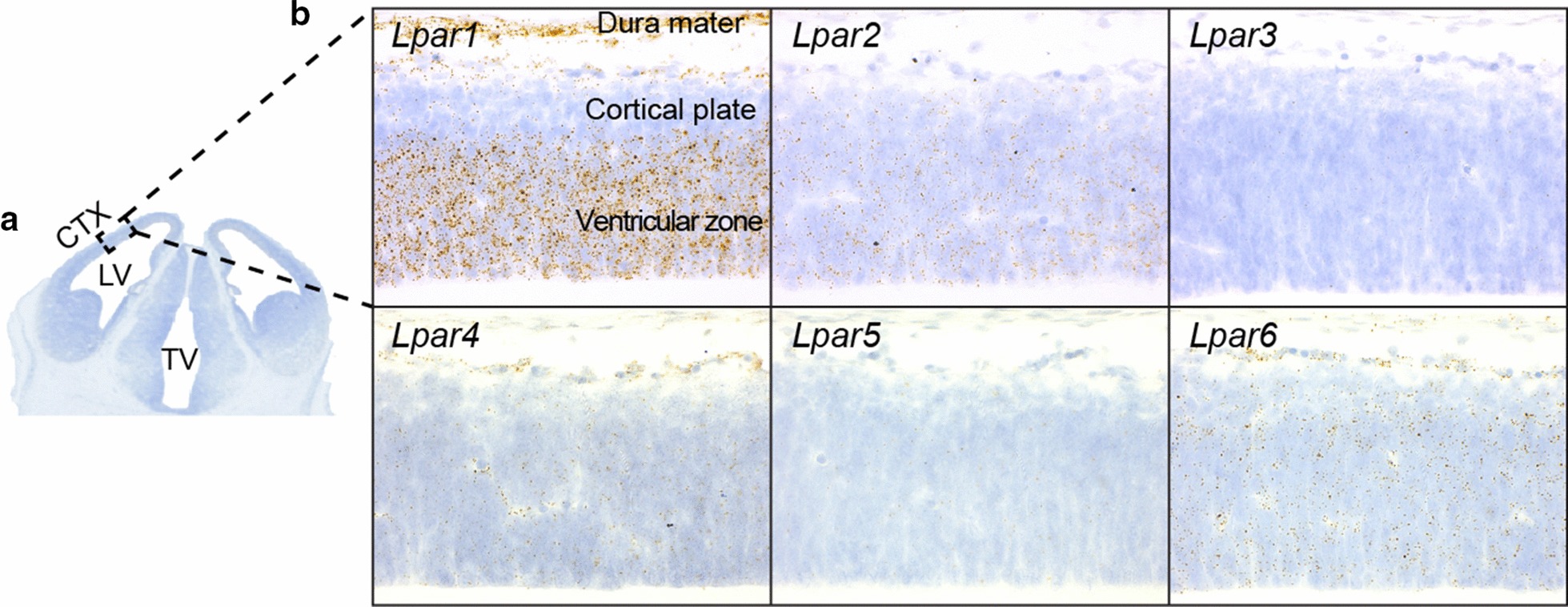
LPA receptor mRNA and total RNA expression in E13.5 cortices. **a** Coronal section of E13.5 mouse brain labeled with hematoxylin to identify lateral ventricle (LV), third ventricle (TV) and cortex (CTX). **b** *Lpar* mRNA expression at E13.5 visualized using RNAscope in situ hybridization. *Brown* and *black puncta* indicate GPCR gene expression in the cortex. Scale bar 20 µm

A recent study examined *Lpar1, Lpar2, Lpar4,* and *Lpar6* mRNA and protein expression in mouse brains aged E16 to P30 [46], but was not extended to earlier developmental time periods coinciding with cortical neurogenesis. Another study examined *Lpar1-5* expression using whole-mount in situ hybridization (ISH) in mouse embryos aged E8.5 to E12.5 [47], which confirmed *Lpar1* and *Lpar2* expression during neurogenic periods [48], but lacked resolution of brain layers and structures. Therefore, to confirm *Lpar1* and *Lpar2* expression, and determine whether any other *Lpars* are present at the cortical plate during development, we explored *Lpar1-6* mRNA expression in the E13.5 cortex using RNAscope in situ hybridization co-stained with hematoxylin (Fig. 2a). *Lpar1* was highly expressed in the ventricular zone, whereas *Lpar2* was diffusely expressed in the ventricular zone and post-mitotic cortical plate. *Lpar3* and *Lpar5* expression was not detected, and *Lpar4* and *Lpar6* mRNA was sparsely expressed throughout the cortical plate (Fig. 2b). The locations of *Lpar1* and *Lpar2* mRNA suggest that these receptors may be the primary mediators of the LPA-induced effects of cleavage plane and fate in progenitor cells.

### LPA signaling shifts the mode of division from proliferative to neurogenic for cells in culture

**Fig. 3:**
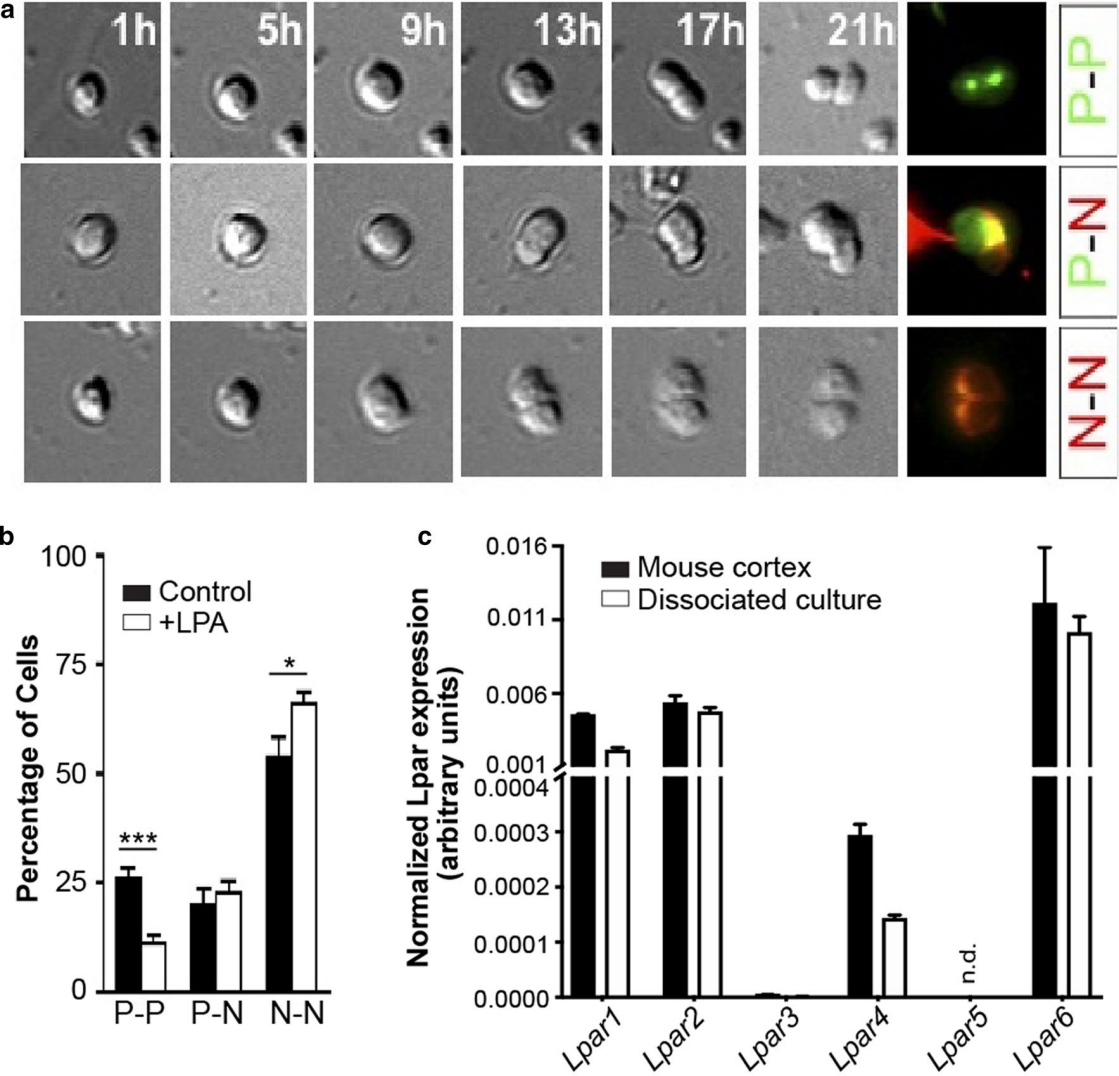
LPA shifts the mode of division from proliferative to neurogenic. **a** Examples of progenitor cells giving rise to three types of daughter-cell pairs. Mitotic division of progenitors dissociated from E13.5 cerebral cortex was followed using time-lapse DIC imaging for 20 h. Daughter-cell fates were identified by immunolabeling progenitor cells with nestin (green) and neural progenitor cells with Tuj-1 (red). P–P: two progenitors; P–N: a progenitor and a neuron; N–N: two neurons. **b** Percentage of cells that underwent mitosis with P–P, P–N and N–N division during the time-lapse imaging. Methods: cortical cells were cultured in serum-free dissociation culture medium or 1 µM LPA-containing serum-free medium for 20 h. Data represents mean + S.E.M. (*n* = 7 replicate experiments, *P = 0.045, ***P = 0.00028, unpaired *t* tests). **c** QPCR-based expression levels of *Lpar1-6* in E13.5 cortex and dissociated cortical cells after 20 h of culture in the control medium. Data are normalized to β-actin expression levels and shown as mean + S.E.M. (*n* = 3). Significance was analyzed by ordinary two-way ANOVA with Sidak’s multiple comparisons test. No significant differences were identified. n.d., not detected

The cell-intrinsic consequences of LPA-induced non-vertical cleavage on proliferative and neurogenic division were determined using cortical cells dissociated from E13.5 mice. Cells were cultured for 20 h in the presence or absence of LPA under time-lapse DIC imaging, and then immunolabeled for Nestin (a progenitor marker) and Tuj-1 (a marker for young post-mitotic neurons) to distinguish daughter cell identities. The cells that underwent mitosis were categorized into three groups: cells that divide into two progenitors (P–P), cells that divide into one progenitor and one neuron (P–N), and cells that divide into two neurons (N–N) (Fig. 3a). LPA treatment significantly reduced P–P division from 26.0% of cells to 11.0% and increased N–N division from 53.7% of cells to 66.1% (Fig. 3b). Therefore, LPA signaling shifted the mode of progenitor division from proliferative to neurogenic, which is in agreement with the induction of non-vertical cleavage planes and the enhanced production of intermediate neural progenitor cells observed in vivo (Fig. 1).

To assess any potential effect of in vitro cell culture on LPA receptor RNA expression, quantitative real-time PCR (qPCR) was performed on cortical cells isolated directly from the brain and on cells cultured for 20 h in serum-free medium. qPCR data revealed that *Lpar1*, *Lpar2*, *Lpar4,* and *Lpar6* were expressed in both freshly isolated cortical cells and in cultured cortical cells, with *Lpar3* and *Lpar5* RNA not significantly expressed in either condition (Fig. 3c). These results are in agreement with the RNAscope data and suggest that no significant LPA mRNA expression changes occurred with short-term culture.

### LPA signaling disrupts adherens junctions (AJs) and cell polarity

**Fig. 4:**
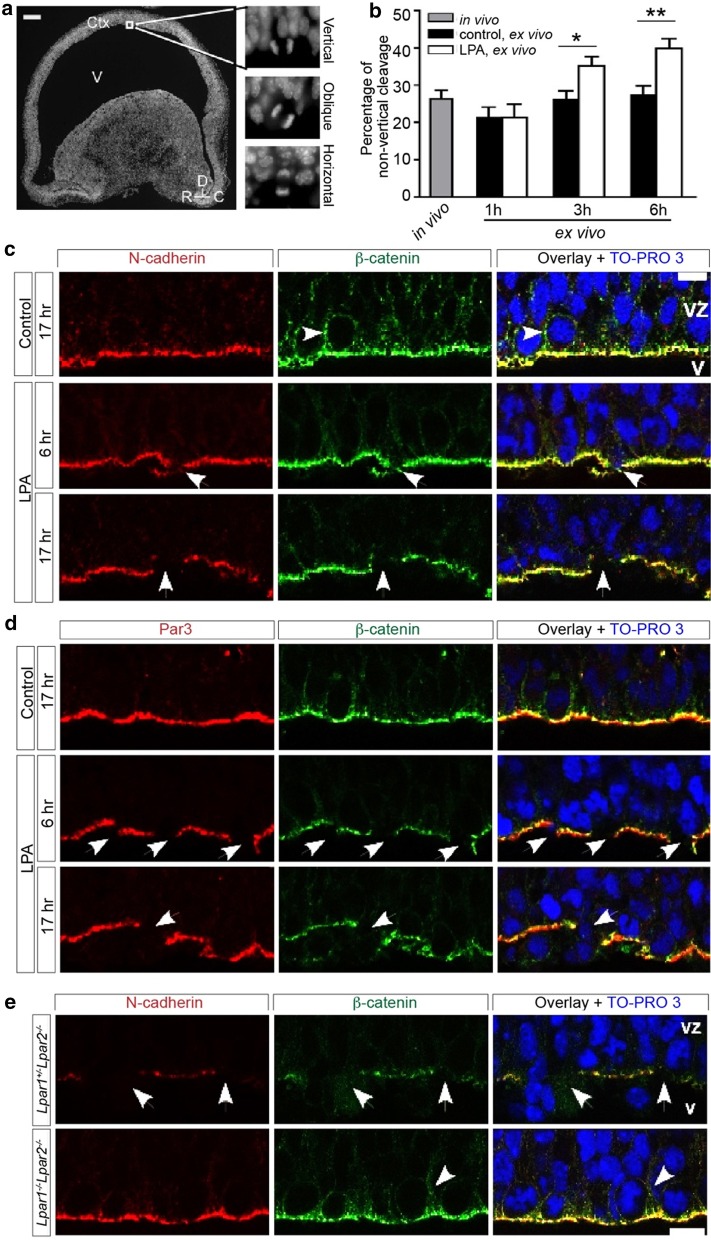
LPA signaling disrupts adherens junctions (AJs), basolateral cell–cell adhesion and cellular polarity. **a** Example of apical mitotic cells with three cleavage plane orientations. A sagittal section from an E13.5 cortical hemisphere stained with DAPI. Apical progenitors at anaphase were classified into three groups according to the angle of mitotic cleavage plane relative to the ventricular surface: vertical, 60°–90°; oblique, 30°–60°; and horizontal, 0°–30°. Ctx, cortex; V, ventricle; R, rostral; D, dorsal; C, caudal. Scale bar 200 µm. **b** Percentage of apical mitotic cells with non-vertical (oblique or horizontal) cleavage planes in vivo following vehicle (control) or LPA treatment. E13.5 cortical hemispheres were freshly fixed or cultured ex vivo in vehicle (control) or 1 µM LPA-containing medium for 1–6 h. Data represent mean + S.E.M. (*n* = 4) in vivo, *n* = 4–5 matched pairs (control vs. LPA), *P = 0.0389, ***P = 0.0072, unpaired *t* tests). **c** Neuroepithelial cells in cortical sections double-immunolabeled with N-cadherin (red), and β-catenin (for basolateral cell-to-cell adhesion, green) with nuclei labeled using TO-PRO-3 (blue). E13.5 cortical hemispheres were cultured ex vivo for 6 or 17 h in control or 1 µM LPA-containing medium. LPA-treated cortices displayed rough apical surfaces with disruption of N-cadherin and β-catenin structures at the apical side (arrows) and lower β-catenin immunoreactivity in basolateral cortex, compared to controls (arrowhead). **d** Cortical sections double-immunolabeled for β-catenin (green), Par3 (for cell polarity, red) and TO-PRO-3 (blue). LPA treatment disrupts Par3 structure at the apical side (arrows). Disrupted sites of Par3 and β-catenin completely overlap. **e** Effects of LPA on AJ disruption in *Lpar1*^+/−^*Lpar2*^−/−^ vs. *Lpar1*^−/−^*Lpar2*^−/−^ cortex. E13.5 cortical hemispheres from *Lpar1*^+/−^*Lpar2*^−/−^ and Lpa_1_^−/−^Lpa_2_^−/−^ littermates were cultured ex vivo for 17 h with or without 1 µM LPA. Exogenous LPA disrupts structures of neuroepithelial cells labeled with N-cadherin (red) and basolateral cell-to-cell adhesion detected by β-catenin (green) at AJs (arrows) and basolateral cortex (arrowhead) in *Lpar1*^+/−^*Lpar2*^−/−^ cortex but not in the *Lpar1*^−/−^*Lpar2*^−/−^ littermate cortex. **c–e** Nuclei were counterstained with TO-PRO 3; representative images analyzed by confocal microscopy are shown. *VZ* ventricular zone, *V* ventricle; scale bar 10 µm

LPA exposure disrupts the radial orientation of cells in the ventricular zone [49], and the disruption of AJs results in cleavage plane randomization during epidermal development [13]; this suggests that LPA signaling may alter cleavage plane orientation by disrupting cellular adhesion. To determine the cell-intrinsic effect of LPA on ependymal layer integrity, we used an ex vivo culture system that simulates the in vivo environment, preserves neurogenesis and enables exogenous ligand exposure to the cortex (Fig. 4a) [36, 50]. We confirmed that ex-vivo culture of E13.5 cortices exhibit enhanced non-vertical cleavage in the presence of LPA. The percentage of cells dividing non-vertically significantly increased within three hours of exposure to LPA, compared to vehicle controls (Fig. 4b). The results suggest that ex-vivo culture recapitulates some of the in vivo markers of LPA signaling present in the ventricular zone.

The structural integrity of the ependymal layer after LPA exposure ex vivo was assessed by immunolabeling for two major AJ components, N-cadherin (a major cadherin expressed in the neuroepithelial cells) and β-catenin (a basolateral cell-to-cell adhesion protein). In control animals, both N-cadherin and β-catenin were concentrated at the AJ on the apical side, and the apical surface remained smooth even after 17 h of ex vivo culture (Fig. 4c, top). In contrast, the apical surface of LPA-treated cortices appeared rough, with several breakages of both N-cadherin and β-catenin structures on the apical side, indicating AJ disruption (Fig. 4c, middle/bottom, arrow). In addition, the level of β-catenin immunoreactivity in the basolateral cortex was lower in LPA-treated cortex compared to controls, indicating disruption of basolateral cell–cell adhesion as well (Fig. 4c, arrowhead). Disruption of AJs and basolateral cell–cell adhesion by LPA was present at 6 h (when cleavage plane alteration is observed) but more pronounced at 17 h. A similar pattern of denudation was observed with cell polarity protein Par3 immunolabeling and double-immunolabeling revealed a completely overlapping disruption of apically localized Par3 and β-catenin (Fig. 4d, middle/bottom, arrow). LPA-mediated AJ disruptions were apparent in the cortex of *Lpar1*^+/−^*Lpar2*^−/−^ mice and absent from the cortices of *Lpar1*^−/−^*Lpar2*^−/−^ littermate controls, demonstrating that the disruptions are LPA_1_ and LPA_2_ receptor-dependent phenomena (Fig. 4e). These data suggest that LPA signaling disrupts AJs, which leads to apical progenitor cell polarity abnormalities.

### AJ disruption randomizes cleavage plane orientation.

**Fig. 5:**
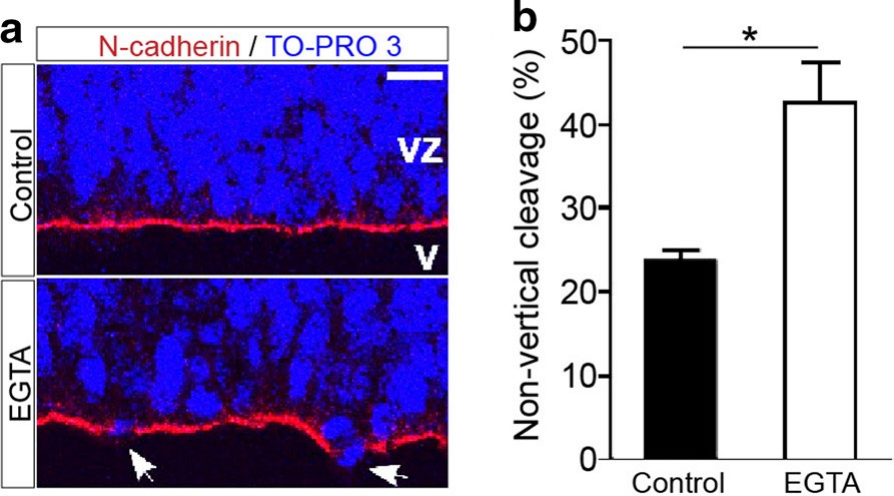
AJ disruption enhanced non-vertical cleavage plane orientation. **a** Cortical sections immunolabeled for N-cadherin (red) to label neuroepithelial cells and counterstained with TO-PRO-3 (blue) to label nuclei following 2 mM EGTA treatment. E13.5 cortical hemispheres were exposed to control or 2 mM EGTA-containing medium for 10 min. EGTA-treatment disrupted N-cadherin structure at the apical side (arrows). Nuclei were counterstained with TO-PRO 3. Representative images, analyzed by confocal microscopy, of 3 paired cortices are shown. CP, cortical plate; VZ, ventricular zone; V, ventricle; scale bar, 10 µm. **b** Percentage of apical mitotic cells with a non-vertical cleavage plane. Data represent mean + S.E.M. (*n* = 3–4, *P = 0.0235, unpaired *t* tests)

LPA signaling altered cleavage plane orientation (Fig. 1d) and disrupted AJs (Fig. 4); however, it is possible that these were independent phenomena. To determine if AJ disruption in the developing cortex leads to cleavage plane alteration, ex vivo cortical hemispheres were incubated with 2 mM EGTA to chelate calcium in the medium and disrupt calcium-dependent cell–cell adhesion. EGTA treatment longer than 10 min severely disrupted AJs and cortical lamination and displaced mitotic cells away from the ventricular surface (data not shown). We therefore analyzed the cortex within 10 min of EGTA treatment, which produced slight breaks of N-cadherin structure on the apical side of EGTA-treated cortex (Fig. 5a). Although there was only slight AJ disruption, non-vertical cleavage plane orientation was significantly enhanced (43.3% ± 4.5) compared to controls (24.5% ± 1.35) (Fig. 5b). It should be noted that in LPA and EGTA-treated cortices, cleavage plane orientation was affected at locations where no obvious AJ disruption was observed, suggesting that subtle AJ disruptions, undetectable by immunohistochemistry, may be sufficient to alter cleavage plane orientation. This result, together with the LPA-induced disruption of AJs (Fig. 4d), demonstrates that abnormal cell–cell adhesion and cell polarity precede cleavage plane orientation abnormalities.

### LPA exposure increases somatic genomic mosaicism (SGM) within neural progenitor cells

**Fig. 6:**
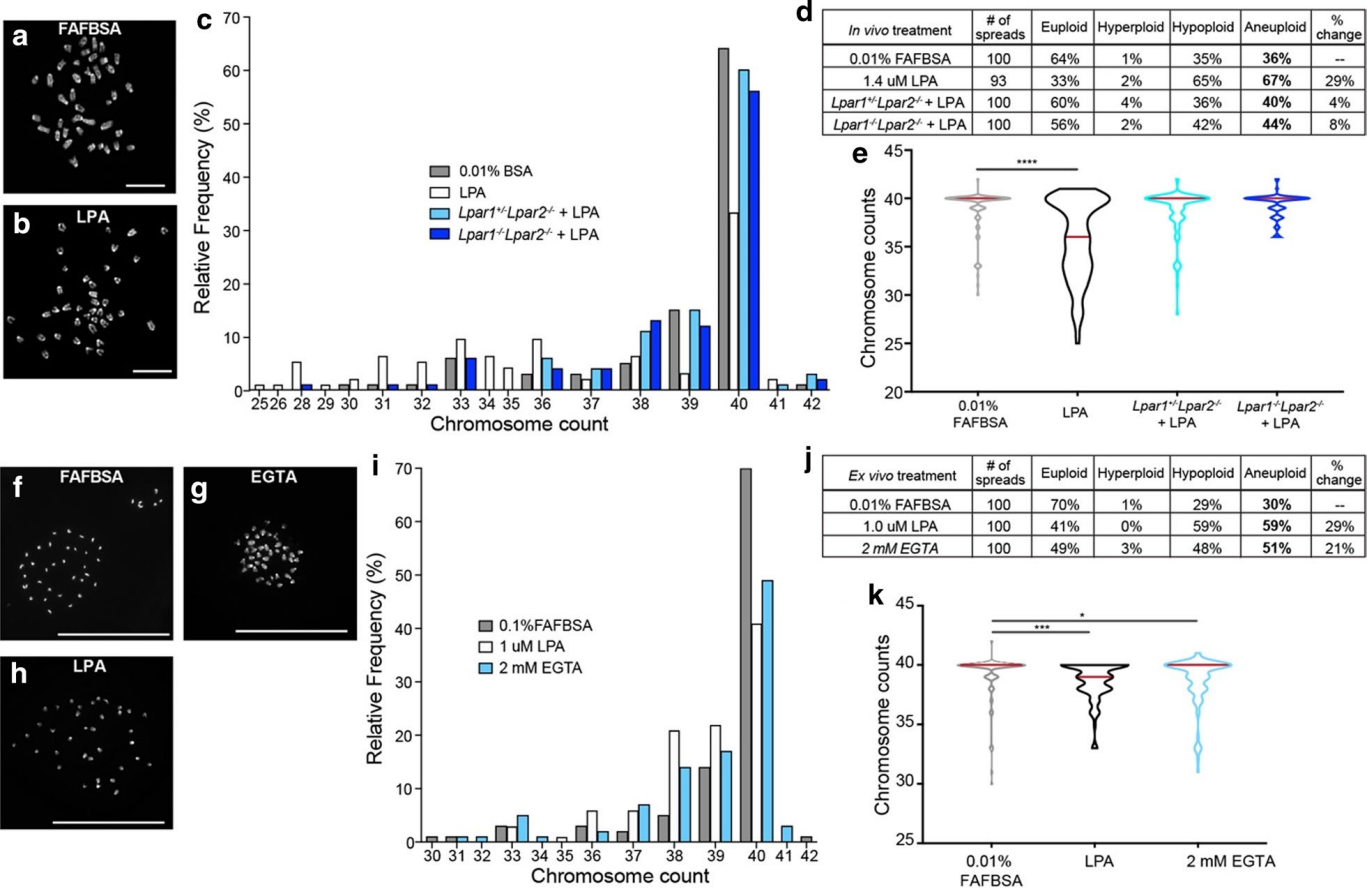
LPA signaling and AJ disruption enhances aneuploidy. **a–e** Chromosomal content from metaphase cells from E13.5 cortices injected with 0.01% FAFBSA (gray), 1.4 µM LPA (white), *Lpar1*^+/−^*Lpar2*^−/−^ + LPA (pale blue), and *Lpar1*^−/−^*Lpar2*^−/−^ + LPA (blue), assessed 6 h after injection **a**, **b**, Representative images of metaphase chromosomes stained with DAPI after in vivo exposure to (**a**) 0.01% FAFBSA (control) or **b** 1.4 μM LPA. Scale bar 10 μm. **c** Frequency of chromosome content in metaphase cells from E13.5 cortices assessed 6 h after injection. **d** Table of total counts and percentages; 100 metaphase spreads were counted for each treatment group. Euploidy = 40 chromosomes, Aneuploidy ≭ 40 chromosomes, Hyperploidy > 40 chromosomes and Hypoploidy < 40 chromosomes. **e** Violin plots of chromosome counts, assessed 6 h after injection. Red line denotes the median. Significance determined by nonparametric Kruskal–Wallis test (Kruskal–Wallis statistic = 45.80, P < 0.0001) and Dunn’s post hoc multiple comparisons correction (****P < 0.0001). **f–k** Chromosomal content from metaphase cells from E13.5 cortices exposed to 0.01% FAFBSA (gray), 1 µM LPA (white), 2 mM EGTA (blue) for 12-h in ex vivo culture. Representative images of metaphase chromosomes stained with DAPI from **f** 0.01% FAFBSA (control), **g** 1 μM LPA and **h** 2 mM EGTA ex vivo exposure. Scale bar, 50 μm. **i** Frequency of chromosome content in metaphase cells from E13.5 cortices exposed for 12-h in ex vivo culture. **j** Table of total counts and percentages; 100 metaphase spreads were counted for each treatment group. **k** Violin plots of chromosome counts. *Red lines* denote the median. Significance determined by nonparametric Kruskal–Wallis test (Kruskal–Wallis statistic = 16.12, P < 0.0003) and Dunn’s post hoc multiple comparisons correction (***P < 0.001 and *P < 0.022). At least 3 brains were assessed per group

Aneuploidy can be a consequence of mitotic spindle abnormalities and altered cleavage plane orientation [26, 51–53]. Studies in mouse models of LPA-induced hydrocephalus [38, 43] and schizophrenia [41] suggest the involvement of genomic mechanisms in disease ontologies [54–58]. To interrogate the consequences of LPA-induced cleavage plane orientation on the genome of individual NPCs, the chromosome complement of E13.5 metaphase cells was assessed 6 h after LPA exposure. LPA exposure increased the proportion of dividing cells with abnormal chromosome counts (67% aneuploid), with the primary effect being a reduction in chromosomal content (65% hypoploid) within 6 h of LPA exposure (Fig. 6a–d). This effect was reversed in both *Lpar1*^+/−^*Lpar2*^−/−^ and *Lpar1*^−/−^*Lpar2*^−/−^ mice with an average of 38.7 ± 0.24 chromosomes and 39.3 chromosomes (Fig. 6c, d), respectively, indicating that enhanced non-vertical cleavage plane frequency is associated with increased aneuploidy in dividing cells, and implicating LPA signaling in the generation of SGM. Notably, genomic changes occurred at the time-point when adherens junctions and cell polarity markers are disrupted along the ependymal layer. To determine the effect of disrupting cell-to-cell adhesion on aneuploidy during cortical development, aneuploidy counts were assessed from ex vivo cortices exposed to 1 μM LPA, 2 mM EGTA, or 0.1% FAFBSA. LPA exposure ex vivo enhanced hypoploidy at a similar rate (59%) to LPA exposure in vivo (65%). Disruption of Ca^2+^ cell–cell adhesion with EGTA also enhanced the frequency of aneuploidy (51%) compared to the vehicle control (29%) (Fig. 6e–i).

## Discussion

This study identified LPA GPCR-signaling as a new mechanism influencing NPC cleavage plane orientation, early cell fate, ependymal layer integrity, and the genome. LPA signaling through LPA_1_ and LPA_2_ increased non-vertical cleavage of apical progenitor cells and enhanced progenitor differentiation into intermediate progenitor cells. Exogenous LPA exposure rapidly disrupted cell–cell adhesion at AJs and at the basolateral cortex prior to neuronal differentiation and showed receptor dependency for *Lpar1* and *Lpar2*, that also resulted in an increase of aneuploid neurons. Cleavage plane orientation, neural cell fate, and the NPC genome were influenced by the loss of LPA signaling through genetic deletion or pharmacological inhibition of its receptors, and by increased LPA signaling induced by exogenous LPA exposure.

### GPCR-mediated signaling alters cleavage plane orientation and results in premature neurogenesis

The effect of LPA signaling on NPC cleavage plane orientation and progenitor cell fate implicates LPA as a critical mediator in cortical development and disease, influencing cell division. LPA is known to promote cytoskeletal changes that include microtubule rearrangements [59, 60] and this role may underlie the abnormal spindle positioning and the resulting cleavage plane orientation in NPCs after LPA exposure. LPA signaling also enhances intermediate progenitor Tbr2+ cell populations, suggesting that LPA-induced cleavage plane abnormalities may trigger an increase in, and displacement of, Tbr2+ cell types. Consistent with this possibility, *Lpar1*-null mice exhibit smaller brain sizes with reduced cortical wall thicknesses [33, 61], which may be due to aberrant progenitor cell cleavage plane orientation and, subsequently, an abnormal mix of cell types or states within the cortex. Enhanced LPA signaling through LPA_1_ and LPA_2_ has been shown to decrease programmed cell death and enhance neurogenesis, pointing to increased Tbr2+ cells as being evidence of cells averting cell death, including through premature cell cycle exit and neuronal differentiation [36].

### Disruption of cell–cell adhesion by LPA leads to altered cleavage plane orientation

Ex vivo cultures demonstrated that cell–cell adhesion at AJs and the basolateral cortex was disrupted by LPA treatment (Fig. 4a) and that disruption of calcium-dependent adhesion by EGTA treatment randomized cleavage plane orientation (Fig. 5). LPA signaling disrupts calcium-dependent conduction prior to cell responsiveness to neurotransmitters like GABA and glutamate [62] and calcium [63], with fluctuations leading to changes in cell–cell adhesion, proliferation and differentiation of progenitor cells [64–66]. Taken together, these results suggest that disruption of cell–cell adhesion by LPA modulation of calcium signaling leads to changes in cleavage plane orientation. Structurally, at least two potential mechanisms may be involved: (1) structures capturing the mitotic spindle at the proper cortical position are disrupted and/or (2) cell polarity is disrupted. Mitotic spindles are recruited to the cellular cortex via two proteins—the basolateral LGN protein and the microtubule-associated protein, NuMA—where LGN recruits mitotic spindles to the lateral cortex via interaction with NuMA [6, 67, 68]. It has been shown that disruption of AJs can alter the localization of LGN/NuMA and randomizes cleavage plane orientation [13] in epidermal development: whether this extends to cerebral cortical development remains an area of future study. It is notable that cadherin expression can be disrupted by LPA signaling [36, 43, 69] with relevance to brain disease conditions of hypoxia [69] and hemorrhage that induces hydrocephalus [43].

The subsequent disruption of cell polarity is also possible. An in vitro study has shown that Par3 tethers microtubules through association with dynein [70]. In LPA-treated cortices, apical Par3 and β-catenin positioning was disrupted (Fig. 4b), suggesting that AJ disruption subsequently perturbed apical Par3 localization or cell polarity. It is plausible that without adjacent cell interactions, both a normal apical-basal polarity and the ability to rotate randomly are not maintained and, as a consequence, cleavage plane orientation would appear randomized even though the spindle is properly recruited to the cortex. It is also possible that apical localization of Par3 might be required for proper cortical spindle capture. The significance of AJ and/or cell polarity in cell fate specification is controversial. In some studies, disruption of AJ and/or cell polarity seems to promote neuronal differentiation [71, 72], while no effect [73, 74] or proliferative effects [75] have been observed in other studies. Thus, we cannot exclude the possibility that disruption of AJ and cell polarity by LPA is not the cause of changes in cleavage plane but simply an associated phenomenon.

### LPA signaling promotes aneuploidy and SGM to produce long-term genomic changes

Enhanced LPA signaling, particularly in disease settings that involve blood exposure producing increased LPA concentrations [40, 41, 43], and hypoxia [69] that produces LPA receptor potentiation, may underlie multiple neurodevelopmental diseases, including hydrocephalus and neuropsychiatric disorders. However, an explanation for the life-long consequences arising from these early developmental insults remains unclear. LPA signaling effects on the genome may provide a partial explanation. Effects could manifest through altered programmed cell death that differentially affects forms of aneuploid cells during development [20] to alter SGM within the developing brain. Notably, the propensity towards chromosome loss is consistent with prior reports that cover a range of technologies including in situ hybridization, single-cell sequencing, and metaphase spreads that have all identified primarily hypoploid NPCs and neural cells during neuronal development [2, 24, 76]. Hyperploid cells clearly are generated and can persist in the adult brain [2, 23, 77] but may show differential prevalence through additional chromosomal loss produced by tripolar mitoses, lagging chromosomes and micronuclei [1], and elimination mechanisms [20]. Aneuploidy, as well as other forms of SGM in neurons, could manifest later in life since they can be integrated within brain circuitry [23, 78, 79]. Genomic alterations constituting SGM within individual brain cells are found at all ages of life in multiple species [24, 80, 81]. Pathophysiologically, aneuploid cells are associated with poor outcomes for multiple diseases [26, 82–86] and thus their increased production following LPA exposure is consistent with increased and sustained disability in brain disease phenotypes.

## Concluding comments

Our data add to the established importance of cleavage plane orientation on NPC fate through identification of an external signaling lipid, LPA, that influences cleavage plane orientation and the genome in a receptor-dependent fashion, through the generation of aneuploid cells. These data indicate that SGM that is present throughout the normal and diseased brain [18] can arise not only through stochastic cell-autonomous processes [2, 87] but also through defined extracellular signals like LPA, to affect aneuploidy and likely other forms of SGM, including smaller CNVs and SNVs [24, 88]. It is therefore likely that the spectrum of SGM forms observed amongst brain cells [18] could be influenced by many yet-to-be defined signals that are critical for brain function, including genomic effects, gene transcription, and neural activity, which have been implicated in somatic gene recombination [88–90], influencing both the normal and diseased brain.

## Data Availability

The datasets used and/or analyzed in the current study are available from the corresponding author upon request.

## Abbreviations

SGM: Somatic genomic mosaicism
NPC: Neural progenitor cell
LPA: Lysophosphatidic acid
Lpar: LPA receptor
RGP: Radial glial progenitor
AJ: Adherens junctions
CNV: Copy number variation
SNV: Single nucleotide variation
GPCR: G protein-coupled receptor
E: Embryonic day
